# Using the Prevention Impacts Simulation Model to Estimate Long-Term Impacts of Multisector Community Partnerships’ Efforts to Address Social Determinants of Health

**DOI:** 10.5888/pcd20.220327

**Published:** 2023-07-20

**Authors:** Amanda A. Honeycutt, Benjamin Yarnoff, Zohra Tayebali, LaShawn Glasgow, Karen Hacker

**Affiliations:** 1 RTI International, Research Triangle Park, North Carolina; 2 Evidera, Bethesda, Maryland; 3 National Center for Chronic Disease Prevention and Health Promotion, Centers for Disease Control and Prevention, Atlanta, Georgia

## Abstract

Public health plays a key role in addressing social determinants of health (SDOH) through multisector community partnerships (MCPs), which contribute to community changes that promote healthy living; however, little is known about the longer-term impact of MCP-driven interventions. We used the Prevention Impacts Simulation Model (PRISM) in a rapid evaluation to better understand the implementation and potential impact of MCPs’ SDOH initiatives. Results suggest that, if sustained, initiatives implemented by the 27 included MCPs may prevent 880 premature deaths and avert $125.7 million in medical costs over 20 years. As a validated model that estimates impact by using available implementation data, PRISM is a useful tool for evaluating SDOH initiatives.

**Summary**

**What is already known on this topic?**

Public health plays a key role in addressing social determinants of health (SDOH), including supporting multisector community partnerships (MCPs); however, little is known about the longer-term impact of MCPs’ SDOH initiatives.

**What is added by this report?**

System dynamics modeling is an underused tool for informing and refining public health interventions. This report demonstrates how the Prevention Impacts Simulation Model (PRISM) can be incorporated in evaluations to estimate cumulative longer-term impacts of SDOH initiatives.

**What are the implications for public health practice?**

If sustained, the initiatives we studied could avert hundreds of deaths and avoid half a billion dollars in costs over 20 years. As a validated model that estimates impact using available implementation data, PRISM is a useful tool for rapid evaluation of SDOH initiatives.

## Objective

Chronic diseases are leading causes of illness, death, and health care costs in our nation (1–3). To reduce the chronic disease burden, it is essential to address underlying social determinants of health (SDOH) (4). However, addressing SDOH is challenging; it requires various intervention approaches across multiple sectors (2,4). Public health’s role in addressing SDOH includes supporting multisector community partnerships (MCPs), but little is known about the longer-term impact of the interventions MCPs promote (5,6).

Improving Social Determinants of Health — Getting Further Faster (GFF) is a rapid retrospective evaluation designed to better understand the implementation and outcomes of 42 MCPs’ SDOH initiatives in 5 domains: 1) built environment (BE), 2) community–clinical linkages (CCL), 3) food and nutrition security (FNS), 4) social connectedness (SC), and 5) tobacco-free policies (TFP). The Centers for Disease Control and Prevention’s National Center for Chronic Disease Prevention and Health Promotion (NCCDPHP) selected these domains based on their links to chronic disease. Although the NCCDPHP framework consists of a more-focused set of SDOH domains than other frameworks commonly used in public health, such as the Healthy People 2030 SDOH Framework (7), it is well-aligned with broader conceptualizations of SDOH. For example, NCCDPHP’s built environment, social connectedness, and community–clinical linkages domains are components of Healthy People 2030’s neighborhood and built environment, social and community context, and health care access and quality domains, respectively (8).

NCCDPHP launched GFF in partnership with the Association of State and Territorial Health Officials (ASTHO) and the National Association of County and City Health Officials (NACCHO). ASTHO and NACCHO conducted a competitive application process to select 42 MCPs from almost 100 applicants. Selection criteria included past success in implementing interventions in 1 or more of the 5 NCCDPHP SDOH domains and partnerships with local or state health departments. ASTHO and NACCHO also contracted with RTI International to conduct the rapid retrospective evaluation, which included virtual discussions with GFF partnerships, document review and abstraction of outcomes data, and simulation modeling. Additional GFF details are in Glasgow et al (9). This brief focuses on simulation modeling of MCP-driven interventions. Because rapid evaluations preclude long-term data collection, we used the existing cardiovascular disease (CVD)-focused Prevention Impacts Simulation Model (PRISM) to estimate the cumulative longer-term impacts of MCPs’ interventions.

## Methods

We used PRISM to estimate the potential impacts of selected MCPs’ initiatives on CVD events, deaths, and medical and productivity costs. PRISM is a system dynamics model that simulates the impacts and costs of 32 strategies to improve CVD-related health behaviors and outcomes; other publications describe the model in detail (10,11). Prior projects have used PRISM to support strategic planning for chronic disease prevention and to evaluate the potential longer-term impacts of community-level strategies to address chronic disease risk factors (eg, tobacco use, obesity, limited access to clinical services) (12,13). The strategies included in PRISM align closely with GFF focus areas. For example, CCL strategies in PRISM address hypertension, diabetes, and high cholesterol management through the provision of high-quality clinical care.

To incorporate PRISM analyses, we first reviewed the 42 MCPs’ GFF applications and identified which partnerships had implemented interventions in 1 or more of the 32 PRISM strategy areas (Table). For example, the mobile farmers markets intervention aligns with the PRISM strategy Fruit and Vegetable Access. Some interventions, such as outdoor smoking bans, are not modeled in PRISM and were therefore not included in our analysis. We asked MCPs to confirm that they had implemented the interventions we had assigned to them; we also requested data on the implementation start date and number of people reached for each intervention. For all interventions that could be modeled in PRISM, we calculated the PRISM lever movement based on established PRISM lever-setting processes (15). We modeled all of an MCP’s interventions in a single PRISM analysis run, conducting 1 analysis run for each MCP.

**Table:** Crosswalk of Multisector Community Partnership (MCP) Interventions to Prevention Impacts Simulation Model (PRISM) Interventions and Levers^a^

GFF focus area	MCP intervention	PRISM lever	PRISM intervention	Intervention intensity applied in PRISM^b^
Built environment	Parks	PA access	Parks	Medium
	Safe streets	PA access	Safe street promotions	Low
	Street design, land use, zoning, active transportation policy	PA access	Street design, land use, zoning, active transportation policy	High
	Walking clubs	PA access	Walking/jogging trail	Low
	Walking trails	PA access	Walking/jogging trail	Low
	Childcare playground equipment	PA in childcare	Installation of portable playground equipment	Medium
	PA in schools	PA in school	PA school requirements	High
	Safe routes to school	PA in school	Safe routes to school	Medium
Clinical–community linkages	Pharmacist program	Quality CVD care	Pharmacist intervention	Medium
	High cholesterol self-management	Quality high cholesterol care	Chronic disease self-management programs (high cholesterol)	Low
	Community health workers	Quality high cholesterol care	Community health workers (high cholesterol)	Medium
	Health IT for chronic disease management	Quality high cholesterol care	Health IT (high cholesterol)	Low
	Community health workers	Quality hypertension care	Community health worker model (hypertension)	Medium
	Culturally tailored interventions for chronic disease management	Quality hypertension care	Culturally tailored interventions	Low
	Health IT for chronic disease management	Quality hypertension care	Health IT (hypertension)	Minimal
	Hypertension self-management	Quality hypertension care	Home blood pressure monitoring	High
	Chronic disease self-management	Quality type 2 diabetes care	Chronic disease self-management programs (diabetes)	Low
	Clinical information system with patient registry to track clinical measures and generate performance reports; includes referral mechanism	Quality type 2 diabetes care	Clinical information system with patient registry to track clinical measures and generate performance reports (diabetes)	Low
	Community Health Worker	Quality type 2 diabetes care	Community health workers (diabetes)	High
	Target underserved populations to increase number of people with access to care	Quality type 2 diabetes care	Target underserved populations to increase number of people with access to care (diabetes)	Low
	Social support for chronic disease management	Quality high cholesterol care	Social support from family and friends	Low
Food insecurity	Fruit and vegetable price reduction	Energy-dense food pricing	Fruit and vegetable price reduction	High
	Community garden	Fruit and vegetable access	Community gardens	Low
	Farmers markets	Fruit and vegetable access	Farmers markets and stands	Medium
	SNAP at farmers markets	Fruit and vegetable access	Farmers markets accepting SNAP/EBT, outreach and transportation for farmers markets	Medium
	Community supported agriculture	Fruit and vegetable access	Food hubs	Low
	Healthy vending machines	Fruit and vegetable access	Healthy vending machines	Medium
	Mobile farmers markets accepting SNAP/EBT	Fruit and vegetable access	Mobile farmers markets accepting SNAP/EBT	Medium
	New grocery stores in underserved areas	Fruit and vegetable access	New grocery stores in underserved areas	Minimal
	Nutrition standards and guidelines in childcare	Fruit and vegetable access	Nutrition standards and guidelines in childcare	Minimal
	Salad bars in schools	Fruit and vegetable access	Salad bars in school cafeterias	Medium
	School gardens	Fruit and vegetable access	School fruit and vegetable gardens	Medium
	NACCHO Food Service Guidelines Local Action Institute	Fruit and vegetable access	School nutrition standards	Low
	Farm to foodbank	Fruit and vegetable access	Worksites: farm-to-site programs, healthy food procurement	Low
	Worksites: NACCHO Food Service Guidelines Local Action Institute	Fruit and vegetable access	Worksites: nutrition standards and guidelines in work cafeterias	Medium
	Nutrition education	Fruit and vegetable access	Educational outreach and awareness of food consumption	Medium
	Promotion in school	Fruit and vegetable access	Promotion in school: food service intervention	Medium
Social connectedness	Depression management	Support services for distressed	Referral to community resources	High
Tobacco-free policies	Smoke-free MUH	Smoke-free MUH	Smoke-free MUH	Medium
	Referrals for smoking cessation services	Smoke quit services	Physician sending patient directly to a counselor, increase physician referrals, increase provider contact	Medium
	Proactive quitlines	Smoke quit services	Proactive tobacco quitlines	Low
	Smoking cessation classes	Smoke quit services	Smoking cessation counseling or motivational interviewing	Low
	Telephone- or cell phone–based cessation intervention	Smoke quit services	Telephone- or cell phone–based cessation intervention	Low
	Smoke-free bars	Workplace smoking bans	Smoke-free bars	Medium
	Smoke-free campuses	Workplace smoking bans	Smoke-free campuses	Medium
	Smoke-free restaurants	Workplace smoking bans	Smoke-free restaurants	Medium
	Smoke-free worksites	Workplace smoking bans	Smoke-free workplaces	High

Abbreviations: CVD, cardiovascular disease; GFF, Getting Further Faster; IT, information technology; MUH, multiunit housing; NACCHO, National Association of County and City Health Officials; PA, physical activity; SNAP/EBT, Supplemental Nutrition Assistance Program/electronic benefit transfer.

a Does not reflect the number of MCPs that implemented each intervention; many interventions were implemented by multiple MCPs.

b To develop a list of evidence-based interventions that can move each PRISM lever, we conducted a review of the scientific literature in 2019 and compiled a list of interventions with a scientific evidence base for each lever. We shared these lists with subject matter experts (SMEs) at Centers for Disease Control and Prevention and revised the lists based on SME input. We then compared the lists against recommendations and systematic reviews by the Community Guide, County Health Rankings, and Cochrane Reviews, adding any interventions that were classified as evidence-based by these 3 sources and reevaluating any that were classified as not evidence-based by these sources. We assigned each intervention a categorical intensity that reflects the strength of the intervention: minimal, low, medium, or high. Interventions were assigned to an intensity category based on several considerations: 1) strength of the evidence as determined by the Community Guide, County Health Rankings, or Cochrane Review; 2) number of articles identified in the literature review; 3) consistency of findings in the literature review; 4) strength of effect sizes found in the literature review; and 5) study characteristics, such as type (eg, trial, observational study, meta-analysis) and sample size. We drew this approach from other recent work that has used the RE-AIM (Reach, Efficacy, Adoption, Implementation, and Maintenance) process (14) and adapted the approach for PRISM analyses by assigning each intervention an intensity setting (minimal, low, medium, or high) that would result in behavior changes of 0.5% for minimal intensity, 2% for low, 5% for medium, and 10% for high. The corresponding PRISM lever movements are 0.04, 0.15, 0.35, and 0.70. Interventions are assumed to be additive in moving PRISM levers; PRISM lever movements are capped at the maximum for a given lever. More details are available in the Appendix of the PRISM Reference Guide (https://prism-simulation.cdc.gov/app/cdc/prism/#/resources).

We obtained cumulative results through 20 years for each MCP, then summed results across MCPs. Results are relative to status quo trends. We analyzed impacts on coronary heart disease events, strokes, deaths, medical costs, and productivity costs; analyses did not include intervention costs. The costs and deaths are for CVD and non-CVD conditions resulting from risk factors included in PRISM. All data were obtained in 2021 for interventions that MCPs had implemented in the previous 7 years.

## Results

From application review, we determined that 32 of 42 GFF MCPs had implemented at least 1 SDOH initiative that could be linked to a strategy modeled in PRISM. Applications for the 10 excluded partnerships described only implementing strategies that are not modeled in PRISM, such as providing cancer screening. Twenty-seven of the remaining MCPs provided data on intervention timing and reach for the PRISM analysis. MCPs included in the analysis had delivered interventions that focus on at least 1 of the 5 SDOH domains, with 6 partnerships working on BE, 10 on CCL, 11 on FNS, 1 on SC, and 7 on TFP. Further information on how we translated the efforts of MCPs into PRISM is available in the Appendix.

The Figure displays the aggregate potential impact of the 27 MCPs’ efforts. We estimated that MCPs’ interventions could potentially avert 150 deaths in 5 years, 340 deaths in 10 years, and 880 deaths in 20 years if sustained among the 1.5 million people reached across the 27 partnerships. Impacts on costs are also meaningful; focusing on 10-year results, we estimate that potential health improvements could lead to averted medical costs of $45.4 million and averted productivity costs of $193.7 million. Costs are reported in 2021 dollars and incorporate no discounting. We saw a large increase in the potential health and economic impacts between the 5- and 10-year and the 10- and 20-year results because PRISM is a system dynamics model that incorporates time delays in the impacts of interventions that address SDOH on health outcomes and mortality.

**Figure:**
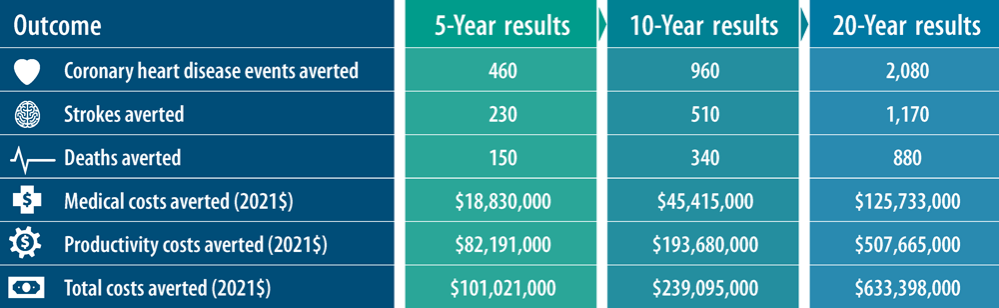
Estimated cumulative potential impacts of efforts implemented by Getting Further Faster partnerships (N = 27) at 5, 10, and 20 years. Coronary heart disease events, strokes, and deaths averted were rounded to the nearest 10. Medical costs, productivity costs, and total costs averted were rounded to the nearest $1,000. Medical costs and productivity costs averted include the costs of cardiovascular disease and risk factors of cardiovascular disease.

**Table:** 

Outcome	5-Year results	10-Year results	20-Year results
Coronary heart disease events averted	460	960	2,080
Strokes averted	230	510	1,170
Deaths averted	150	340	880
Medical costs averted (2021 dollars)	$18,830,000	$45,415,000	$125,733,000
Productivity costs averted (2021 dollars)	$82,191,000	$193,680,000	$507,665,000
Total costs averted (2021 dollars)	$101,021,000	$239,095,000	$633,398,000

## Discussion

PRISM was developed to support planning and evaluation of 32 broad strategies to prevent or manage CVD by addressing CVD risk factors. Leveraging close alignment between PRISM and GFF strategies, we used PRISM to analyze the potential longer-term impacts of SDOH initiatives implemented by 27 MCPs. We found that MCPs’ efforts, if sustained, could potentially avert 880 deaths and $633.4 million in costs over 20 years. Because it can take years for SDOH interventions to have a measurable impact on health outcomes, the average annual impact of MCPs’ interventions increased considerably over time. Within the GFF cohort, the potential average number of deaths per year averted was 1.5 times larger after 20 years of implementation compared with 5 years (44 averted deaths per year versus 30 deaths per year).

Our analysis has limitations. These results likely provide conservative impact estimates for the GFF cohort because some MCPs implemented interventions that are not modeled in PRISM. PRISM focuses primarily on CVD; although it captures costs and deaths from non-CVD conditions that are attributable to CVD risk factors, such as smoking, it does not fully capture impacts on non-CVD conditions. Additionally, the retrospective nature of the evaluation meant that some MCPs were unable to provide the data needed for PRISM analyses for all the interventions that they implemented. However, our estimates may overstate impact if MCPs’ efforts are not sustained with at least the current numbers of people reached. Another limitation is the lack of standardization in intervention implementation across MCPs. Unless all interventions were implemented in a manner that has been shown to be effective, our estimates may overstate the long-term impact.

Despite limitations, incorporating PRISM analysis in our rapid retrospective evaluation provided helpful information about the potential longer-term impact of MCPs’ SDOH initiatives. PRISM analysis was used to overcome common obstacles to MCP-driven intervention evaluation, including the challenges of working within evaluation timeframes that are much shorter than the time required for interventions to yield health and other salient outcomes (5). Our work also helps address a key gap in the literature around the use of modeling to inform decision-making in the public health sector (13). As public health mobilizes to better address SDOH and advance health equity (16), mathematical models like PRISM could be integrated into program planning and evaluation. This could be an excellent accompaniment to other evaluation efforts.

## Appendix Example of Using PRISM to Analyze Long-Term Impacts of MCPs’ Interventions

This appendix provides details on how we translated the efforts of multisector community partnerships (MCPs) into the Prevention Impacts Simulation Model (PRISM). Appendix Table A1 shows, for 2 MCPs (denoted as A and B), their efforts to address social determinants of health (SDOH) factors, their reported reach for each intervention effort, and how we translated those efforts into PRISM lever movements. We indicate the PRISM intervention that our study team assigned to each MCP effort, the PRISM lever affected by that intervention, and the PRISM lever movement associated with the MCPs’ interventions. PRISM levers were not allowed to move beyond the best possible level for a given lever (eg, the percentage of workplaces that allow smoking cannot go below 0, as reflected for MCP B in their efforts to reduce indoor smoking in workplaces). The information in Appendix Table A1 reflects the MCP-specific inputs that were used in the PRISM analysis.

Appendix Table A2 provides intermediate and longer-term results from PRISM for the 2 MCPs shown in Appendix Table A1. The results are based on the PRISM lever movements shown in Appendix Table A1. The baseline mean column shows the mean in 2021 for selected outcomes from PRISM sensitivity analysis runs for the scenarios shown in Appendix Table A1. In 2021, only about 39% of those with diabetes had the condition adequately controlled, 50% of those with high blood pressure had achieved control, and 53% of those with high cholesterol had the condition controlled. About 18% of US adults were smokers. The results in the columns for MCPs A and B depict how the efforts shown in Appendix Table A1 affect the intermediate outcomes in 10 and 20 years, respectively. Because MCP A’s work focuses on managing chronic disease, the disease management outcomes for MCP A improve considerably, whereas those for MCP B show little improvement beyond current trends (eg, expected reductions in adult smoking, even without additional targeted intervention).

Appendix Table A2 also shows that impacts tend to more than double between 10 and 20 years because these interventions take time to affect chronic disease and mortality-related outcomes. Additionally, the higher reach of MCP B means greater impact than for MCP A, despite the large impact that MCP A’s efforts produce per person reached. Our analysis calculated results for each MCP by using the same approach and then summed outcomes across all MCPs to demonstrate the expected impact of these 27 MCPs’ efforts on health-, mortality-, and cost-related outcomes. Ten-year PRISM per capita outcomes aggregated by the numbers reached in each MCP are shown for all 27 MCPs in Appendix Table A3.

**Appendix Table A1:** Two MCPs, Their Interventions Included in the Getting Further Faster Evaluation, and Prevention Impacts Simulation Model (PRISM) Inputs

MCP	SDOH initiative reported by MCP	Reach reported by MCP, no.	PRISM intervention assignment	PRISM lever	Intervention intensity	Initial lever value	PRISM lever movement	PRISM lever value for analysis
A	Community health workers	167	Community health workers (high cholesterol)	Quality high cholesterol care, no previous CVD event	Medium	0.565	0.175	0.74
A	Community health workers	167	Community health workers (high cholesterol)	Quality high cholesterol care, post-CVD event	Medium	0.825	0.175	1.00
A	Community health workers	167	Community health workers (diabetes)	Quality type 2 diabetes care, no previous CVD event	High	0.51	0.35	0.86
A	Community health workers	167	Community health workers (diabetes)	Quality type 2 diabetes care, post-CVD event	High	0.515	0.35	0.865
A	Community health workers	167	Community health worker model (hypertension)	Quality hypertension care, no previous CVD event	Medium	0.685	0.175	0.86
A	Community health workers	167	Community health worker model (hypertension)	Quality hypertension care, post-CVD event	Medium	0.69	0.175	0.865
B	Fruit and vegetable price reduction	1,800	Fruit and vegetable price reduction	Energy-dense food pricing	High	0.01	0.14	0.15
B	Smoke-free bars	1,579	Smoke-free bars	Workplace smoking bans	Medium	0.04	0.14	0
B	Smoke-free campuses	1,579	Smoke-free campuses	Workplace smoking bans	Medium	0.04	0.14	0
B	Smoke-free restaurants	98,012	Smoke-free restaurants	Workplace smoking bans	Medium	0.04	0.14	0
B	Smoke-free workplaces	21,700	Smoke-free workplaces	Workplace smoking bans	High	0.04	0.28	0

Abbreviations: CVD, cardiovascular disease; MCPs, multisector community partnerships; PRISM, Prevention Impacts Simulation Model; SDOH, social determinant of health.

**Appendix Table A2:** Prevention Impacts Simulation Model (PRISM) Output for 2 Multisector Community Partnerships (MCPs) in 10 Years and 20 Years

Outcomes	Baseline mean, 2021	MCP A, events or costs averted		MCP B, events or costs averted	
		10-Year results	20-Year results	10-Year results	20-Year results
**Intermediate outcomes**					
Controlled fraction of diabetes subpopulation	39.2%	78.6%	78.7%	39.7%	40.1%
Controlled fraction of high blood pressure subpopulation	50.0%	74.6%	74.7%	50.2%	50.4%
Controlled fraction of high cholesterol subpopulation	53.3%	71.4%	72.1%	55.2%	56.2%
Smoker fraction of total population	18.3%	15.2%	13.2%	15.2%	13.2%
**Longer-term outcomes**					
Cumulative number of strokes	4.36 per thousand	8.44	18.07	41.27	103.57
Cumulative number of coronary heart disease events	6.86 per thousand	8.39	17.52	82.86	187.00
Cumulative medical costs^a^	$1,070 per person	$513,370	$1,349,290	$3,479,390	$10,468,080
Cumulative productivity costs^a^	$2,950 per person	$2,241,080	$5,641,280	$9,494,090	$28,000,410
Cumulative deaths	5.03 deaths per thousand	4.61	11.12	16.73	47.45

^a^ Cost outputs from PRISM are in 2018 dollars. Costs are shown here in 2021 dollars and were inflated by using the medical component of the Consumer Price Index for medical costs and the Employment Cost Index for productivity costs. Costs are rounded to nearest $10.

**Appendix Table A3:** Ten-Year Results Using Prevention Impacts Simulation Model Output for the 27 Multisector Community Partnerships (MCPs) That Participated in Getting Further Faster

MCP (sorted by impact)	No. of strokes averted	No. of coronary heart disease events averted	No. of deaths averted	Medical costs averted, in 2021 dollars	Productivity costs averted, in 2021 dollars
1	166.52	281.49	135.45	$14,517,284	$70,153,868
2	106.22	136.05	55.66	$7,965,325	$30,589,261
3	20.54	39.40	39.73	$3,125,567	$26,888,457
4	27.15	251.81	23.09	$7,045,020	$15,380,845
5	41.27	82.86	16.73	$3,479,392	$9,494,093
6	24.26	24.85	13.05	$1,502,098	$6,356,518
7	22.96	22.82	12.53	$1,396,739	$6,097,349
8	22.82	20.44	10.98	$1,226,953	$7,102,645
9	12.29	11.29	5.77	$648,997	$3,745,502
10	12.21	18.17	4.97	$941,151	$2,450,398
11	8.44	8.39	4.61	$513,371	$2,241,078
12	7.04	9.15	4.09	$562,064	$2,220,555
13	7.94	7.11	3.82	$427,051	$2,473,194
14	6.15	5.55	2.94	$328,856	$1,900,900
15	5.33	4.79	2.55	$285,487	$1,651,425
16	4.35	8.02	1.78	$265,886	$1,183,909
17	2.76	2.75	1.51	$168,040	$733,566
18	3.65	5.48	1.48	$280,148	$729,432
19	2.96	6.71	1.24	$229,550	$807,657
20	2.08	3.12	0.84	$159,715	$415,856
21	1.52	1.69	0.53	$95,307	$286,469
22	1.10	2.90	0.47	$85,971	$338,805
23	1.10	1.64	0.44	$84,028	$218,786
24	0.28	1.62	0.18	$46,166	$116,825
25	0.37	0.54	0.15	$28,543	$74,351
26	0.05	0.06	0.04	$4,339	$20,719
27	0.03	0.05	0.01	$1,735	$7,733
**Total^a^**	**511**	**959**	**344**	**$45,414,800**	**$193,680,200**

^a^ Totals across all MCPs have been rounded, with costs rounded to the nearest hundreds, and as a result may not exactly match the sum of values for the 27 individual MCPs.
